# Efficacy and safety of electro-acupuncture (EA) on insomnia in patients with lung cancer: study protocol of a randomized controlled trial

**DOI:** 10.1186/s13063-020-04721-4

**Published:** 2020-09-14

**Authors:** Hongyu Yue, Shuang Zhou, Huangan Wu, Xuan Yin, Shanshan Li, Tingting Liang, Yan Li, Zhihong Fang, Xufeng Zhang, Linglin Wang, Min Han, Xiaolei Chen, Ming Zhang, Wei Zhang, Zhangjin Zhang, Shifen Xu

**Affiliations:** 1 Shanghai Municipal Hospital of Traditional Chinese Medicine, Shanghai University of Traditional Chinese Medicine, Shanghai, 200071 China; 2 Acupuncture and Moxibusion College, Shanghai University of Traditional Chinese Medicine, Shanghai, 201203 China; 3 Research Institute of Acupuncture and Meridian, Shanghai University of Traditional Chinese Medicine, Shanghai, 200003 China; 4 Putuo Hospital, Shanghai University of Traditional Chinese Medicine, Shanghai, 200062 China; 5 Xie-Tu Community Health Service Centre, Xuhui District, Shanghai, 200032 China; 6 Shanghai Chest Hospital, Shanghai Jiao Tong University, Shanghai, 200030 China; 7 Department of Biostatistics, School of Public Health, Fudan University, Shanghai, 200032 China; 8 School of Chinese Medicine, The University of Hong Kong, Hong Kong, China

**Keywords:** RCT, Protocol, EA, Lung cancer-related insomnia

## Abstract

**Background:**

Cancer-related insomnia (CRI) is one of the most prevalent complaints among cancer survivors and severely impairs patients’ quality of life. As a popular non-pharmacological alternative treatment, acupuncture provides a good clinical curative effect on insomnia. The aim of this trial is to evaluate efficacy and safety of electro-acupuncture on insomnia in patients with lung cancer.

**Method:**

This is a protocol for a multicenter randomized single-blinded sham-controlled trial. We will randomly assign 252 eligible patients with lung cancer-related insomnia into two groups at a ratio of 1:1, the treatment group (EA) and the control group (sham EA). All treatment will be given 3 times per week for 8 weeks, and a 12-week follow-up will be conducted. The primary outcome will be measured by the Pittsburgh Sleep Quality Index (PSQI). The secondary outcomes will include sleep parameters recorded from the actigraphy, scores from Quality of Life Questionnaire Core-30 (QLQ-C30), and Patient Health Questionnaire-9 (PHQ-9). All adverse effects during the trial will be assessed by the Treatment Emergent Symptom Scale (TESS). All analyses will be based on ITT principle and performed with the statistical software SPSS (version 24.0) by *t* test, rank-sum test, chi-square, and so on. A two-sided significance level will be set at 5%.

**Discussion:**

This large-sample trial protocol will evaluate the efficacy of electro-acupuncture on insomnia in patients with lung cancer. This protocol, if proven to be effective, will contribute to filling the gap in treatment options in the CRI field and provide a promising intervention for insomnia in lung cancer survivors.

**Trial registration:**

ChiCTR ChiCTR1900026395. Registered on 8 October 2019, http://www.chictr.org.cn/showproj.aspx?proj=44068

## Background

Cancer-related insomnia (CRI) is one of the most prevalent complaints among cancer survivors, especially in the lung, breast, head, and neck cancers [1]. Lung cancer has the highest prevalence of incidence and mortality of malignant tumors in China. It is expected that by 2020, the incidence of lung cancer will exceed 800,000 and the death toll will approach 700,000 in China alone [2]. According to Wei’s research [3], the incidents of cancer-related insomnia among lung cancer patients has risen up to 68.4%. Concurrently, foreign researchers [4, 5] have shown that lung cancer patients have a relatively high incident rate of all sleep-related problems when compared with all other cancer types. The causative link between cancer survivors and increased incidents of insomnia remains uncertain and may consist of complex interactions of various factors. Savard and Morin [6] divided the etiology into three main categories: predisposing, precipitating, and perpetuating factors. Different pathological types and clinical stages also have significant effects on CRI as well as symptoms induced by cancer and adverse effects created by anti-cancer therapies. CRI is a frequently overlooked consequence of cancer diagnosis and treatment [7], mainly manifesting as difficulty in initiating sleep or maintaining sleep, waking up earlier than desired, and patient’s resistance to go to bed on an appropriate schedule. It results in negative effects, such as fatigue, attention impairment, irritability, and daytime sleepiness, all of which severely impairs the overall quality of life and even prognosis of cancers [8]. Therefore, lung cancer-related insomnia is an important problem to be solved among the lung cancer survivors.

At present, pharmaceutical therapy is the most common therapy for management of insomnia with lung cancer, such as benzodiazepines (e.g., clonazepam, midazolam) and non-benzodiazepines (e.g., zolpione, zalepron). Although pharmacotherapy shows good short-term curative effect, long-term large doses are not recommended due to drug resistance, psychological dependence, and physical dependence [9]. Meanwhile, the efficacy and safety of its interaction with anti-tumor drugs is not confirmed. Therefore, many patients refuse hypnotics, for they can impair the overall quality of life and even prognosis of tumors. In addition to pharmaceutical therapy, Cognitive Behavioral Therapy (CBT), exercise intervention, and herbal medicine are applied in clinic as primary treatments; however, all lack valid evidence to confirm their efficacy and safety. Therefore, it is imperative to find a safe, effective therapy with few side effects, as currently, there is a dearth of effective methods to treat insomnia related to lung cancer.

Acupuncture has showed an increasing prevalence all over the world, and its definite curative effect is widely recognized by countless patients. As an acknowledged complementary treatment, acupuncture is a non-pharmacological method in treating various common disease, including insomnia, depression, headache, back pain, facial paralysis, and so on. In addition, acupuncture also shows its obvious effect in intractable diseases, such as Parkinson’s disease, cerebral infarction, ankylosing spondylitis, and so on [10]. As a mature technology, eletro-acupuncture device has already been standardized by National Medical Product Administration in China. As a safe and effective treatment method, EA is widely accepted by patients and applied in clinic with the advantages of long-term stimulation, easy to control the intensity of stimulation, and efficacy of enhancing the needling sensation. It can adjust the biological dysfunctional of human body and has the effects of relieving pain, inducing sedation, and promoting the circulation of qi and blood, particularly suitable for insomnia. According to various researches, acupuncture provides a good clinical curative effect on insomnia, takes effect quickly, and is relatively safe with few side effects. Previous studies [10] have provided evidence that acupuncture has effects on sleep disorders in general population, but there are few high-quality studies focused on acupuncture as a method for the treatment of lung cancer-related insomnia. We will take the lead to prove the safety and efficacy of acupuncture as a treatment of insomnia in lung cancer patients. Otte et al. [11] carried out a single group clinical trial, applying actigraphy as the main outcome measurement. After three sessions of acupuncture treatment, sleep duration and sleep efficiency were significantly improved among 10 breast cancer patients. There are two studies on the effects of acupuncture on depression symptoms [12] and hot flashes in cancer survivors [13]. But it is uncertain that acupuncture has the curative effect on CRI, for that sleep-related measurement is only a secondary outcome in these studies. Song et al. [14] have carried out a large-sample randomized controlled trials of acupuncture on treatment of CRI, which divided into two groups: treatment group with acupuncture and control group that received estazolam and no acupuncture. The trial result showed that there were no statistical differences between the two groups in the sleep efficiency during the treating period. However, this trial exhibited some nullifying deficiencies, including such confounding factors as no restrictions to cancer types and no detailed refinement of inclusive criteria and treatment methods [15]. Therefore, rigorous high-quality and well-designed RCTs with large samples are urgently needed to prove the safety and efficacy of acupuncture treatment on insomnia in lung cancer patients. These contributions will lay a solid foundation for further promotion and application of acupuncture in the future.

We therefore designed this large-sample multicenter randomized controlled trial with rigorous methods, effective randomization, accurate statistical analysis, and valid measures. We hypothesized that electro-acupuncture is an effective and safe method in treating insomnia among lung cancer patients. The objectives of this trial are:

1. To evaluate efficacy of electro-acupuncture on insomnia in lung cancer patients as measured by PSQI scores, data from actigraphy, and dose of hypnotics compared with sham EA.
2. To evaluate safety of eletro-acupuncture compared with shame EA by number of adverse events, number of dropout, and the reasons both in treatment group and sham group.
3. To determine whether EA can relieve depression and improve quality of life in lung cancer patients as measured by PHQ-9 scores, QLQ-C30 to compare the between-group difference.

## Methods

### Study design

**Fig. 1:**
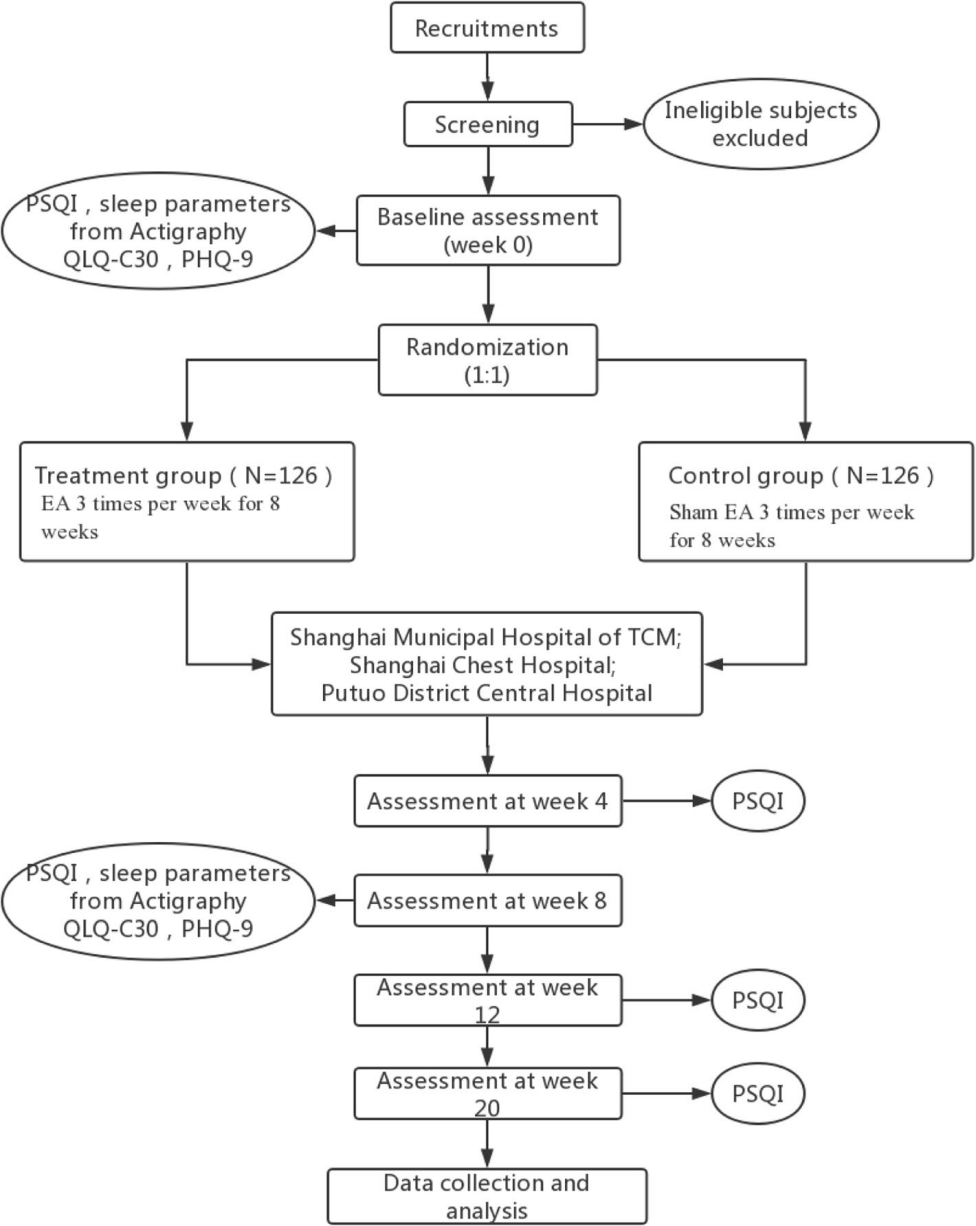
Study flowchart

This is a study protocol of a multicenter randomized patient-and-assessor-blinded sham-controlled clinical trial designed to evaluate the safety and efficacy of electro-acupuncture on insomnia in patients with lung cancer. A total of 252 eligible outpatients and in-patients with lung cancer-related insomnia will be recruited from three hospitals in Shanghai: Shanghai Municipal Hospital of Traditional Chinese Medicine (TCM), Shanghai Chest Hospital, and Putuo District Central Hospital. Each participant will be informed of study-related information and sign a written informed consent before they enter the trial. They will then be randomly divided into treatment group and control group at a ratio of 1:1, receiving EA and sham EA 3 times per week for 8 weeks respectively. The schedule of enrollment, intervention, and assessment is presented in Table 1. This trial is strictly designed to follow the Consolidated Standards of Reporting Trials (CONSORT) statement and Standards for Reporting Intervention in Controlled Trials of Acupuncture (STRICTA) [16] recommendations. The flowchart of the trial is presented in Fig. 1, and the study design schedule is presented in Table 1.

**Table 1:** Study design schedule

Period week(W)	Enrollment	Allocation	Treatment			Follow-up	
	Week − 1	Week 0	Week 1	Week 4	Week 8	Week 12	Week 20
Enrollment	**×**						
Informed consent	**×**						
Medical history	**×**						
Merger disease	**×**						
Randomization		**×**					
Intervention			**×**	**×**	**×**		
Primary outcome							
PSQI	**×**	**×**		**×**	**×**	**×**	**×**
Secondary outcomes							
Actigraphy		**×**			**×**		
QLQ-C30		**×**			**×**		
PHQ-9		**×**			**×**		
Dose of hypnotics		**×**	**×**	**×**	**×**	**×**	**×**
Adverse event monitoring (side effects and complications)	**×**	**×**	**×**	**×**	**×**	**×**	**×**
Assessment of credibility		**×**					
Assessment of blinding success			**×**		**×**

### Participants

#### Recruitment

This multicenter randomized controlled trial will be conducted in Shanghai Municipal Hospital of Traditional Chinese Medicine (TCM), Shanghai Chest Hospital, and Putuo District Central Hospital. We plan to recruit 252 participants in total through online and offline advertisement inside and outside these hospitals. Patients who have interest in entering this trial can phone the researchers or communicate with them face to face for more study details. The researchers will screen patients according to the inclusion and exclusion criteria and then thoroughly inform the patients of benefits gained from this trial and potential adverse reactions. Eligible participants will be proceeded with the intervention and assessments after signing informed consent.

#### Inclusion criteria

1. Male or female patients aged between 19 and 70 years old
2. Diagnosed with stage I–IIIA pulmonary malignancy according to imaging result, histologic examination, and TNM classification
3. Continuous insomnia related to lung cancer treatment or cancer itself for at least 3 months, and meets the diagnostic criteria of chronic insomnia according to The Diagnostic and Statistical Manual of Mental Disorders, Fifth Edition (DSM-V), American Psychiatric Association (APA)
4. An Eastern Cooperation Oncology Group (ECOG) performance status of less than 2
5. Score of Pittsburgh Sleep Quality Index (PSQI) of more than 11
6. Have never received acupuncture treatment
7. Willing to participate in the trial and provide written consent

#### Exclusion criteria

1. A plan for surgery or chemotherapy during the trial
2. A diagnosis of secondary insomnia caused by depression, anxiety or other psychiatric disorders, and addition of caffeine, alcohol, or drugs
3. Index of cancer pain measured by the numeric rating scale ≥ 4
4. A diagnosis of severe cognitive deficit failing to cooperate
5. A diagnosis of severe diseases of the cardiovascular, hepatic, renal, cerebrovascular, or hematopoietic systems
6. Acupuncture area with skin infection, ulcer, and soars
7. Pregnant or breastfeeding women
8. Having participated in other clinic trials within 4 weeks of the beginning of this trial

#### Randomization and allocation concealment

This trial will adopt stratified, variable block randomization method with setting a random block size as 2, 4, or 6. An independent researcher, who has no contact with participants, assessors, and acupuncturists, will use SPSS (version 24.0) to generate random numbers with the randomized blocks, dividing 252 participants into two groups at a ratio of 1:1: treatment group and sham group, and randomly allocate them into three different hospitals: Shanghai Municipal Hospital of Traditional Chinese Medicine (TCM), Shanghai Chest Hospital, and Putuo District Central Hospital. The researcher will make random allocation cards, each with its allocated hospital and group recorded, and seal each card into an opaque envelope, which will not be revealed until the first acupuncture treatment.

#### Blinding

This is a single-blinded (patient-assessor-blinded) study. Before treatment, all participants will be informed that they will be assigned to either EA group or sham EA group. They will also be required to wear eye-patch in a private quiet space during the whole treatment period. The principal researcher, assistant researchers, data analysts, assessors, and statisticians will all be blinded to the group allocation. Only the acupuncturist will know their allocated groups, but they will not be informed of any qualitative information on the patient, including severity of lung cancer-related insomnia, merger disease, or dose of hypnotics. This trial will set up a data safety monitoring committee according to the guidance of Data Safety Monitoring Board (DSMB). Experts in the committee will be responsible for monitoring the data safety and have the right to reveal blinded data.

### Interventions

Participants will receive either EA or sham EA, three times per week for 8 weeks, and the follow-up period will be 3 months. Only a licensed acupuncturist with more than 2 years of clinical experience will be responsible for performing the real and sham acupuncture treatment. All manipulation should adhere to the STRICTA. Every treatment session will last for 30 min in a private quiet space, with each participant wearing an eye-patch and in a lying position.

#### Electro-acupuncture (EA) treatment

Participants in treatment group will receive real electro-acupuncture treatment. Stainless steel 0.25 × 40 mm acupuncture needles (Wuxi Jiajian Medical Material Co., Ltd., Wuxi, China) will be inserted into 15 acupoints, including 11 core acupoints and 4 additional acupoints. The core acupoints are GV20 (Baihui), GV24 (Shenting), GV29 (Yintang), bilateral EX-HN22 (Anmian), HT7 (Shenmen), SP6 (Sanyinjiao), and ST36 (Zusanli), and the additional acupoints should be chosen from the following points: LR3 (Taichong), KI3 (Taixi), PC6 (Neiguan), CV4 (Guanyuan), CV12 (Zhongwan), LR14 (Qimen), ST44 (Neiting), and LR2 (Xingjian), based on syndrome differentiation of participants. The locations, indications, and manipulation methods are specifically presented in Table 2. After the needle is inserted to a certain depth of points, it will be manipulated with needling techniques including lifting and thrusting or rotating methods for Deqi sensation. After Deqi, the electro-acupuncture device (CMNS6-1, Wuxi Jiajian Medical Device Co., Ltd., China) will be applied connecting to points GV20 (Baihui), GV29 (Yintang), bilateral SP6 (Sanyinjiao), and ST36 (Zusanli) with 3-Hz frequency and the varying amplitude depending on the comfort of the participant which will be limited between 2 and 5 mA, to strengthen the needling sensation.

**Table 2:** Location, indication, and methods of acupoints for treating lung cancer-related insomnia

Acupoints	Location	Method
GV20 (Baihui)	5 *cun* directly above the midpoint of the anterior hairline, at the midpoint of the line connecting the apexes of the two auricles.	Subcutaneous insertion 16–26 mm. Moxibustion can be used for reinforcing Yang.
GV24 (Shenting)	0.5 *cun* directly above the midpoint of the anterior hairline.	Subcutaneous insertion 16–26 mm.
GV29 (Yintang)	On the forehead, at the midpoint between the two medial ends of the eyebrow.	Subcutaneous insertion 10–16 mm.
EX-HN22 (Anmian)	At the midpoint between Fengchi and Yifeng, at the middle of sternocleidomastoid tendon.	Perpendicular insertion 33–49 mm.
HT7 (Shenmen)	At the ulnar end of the transverse crease of the wrist, in the depression on the radial side of the tendon of m. flexor carpi ulnaris	Perpendicular insertion 10–16 mm.
SP6 (Sanyinjiao)	3 *cun* above the medial malleolus, on the posterior border of the medial aspect of tibia.	Perpendicular insertion 33–49 mm.
ST36 (Zusanli)	3 *cun* below Dubi (ST35), one finger-breadth (middle finger) from the anterior crest of tibia	Perpendicular insertion 33–66 mm
LR3 (Taichong)	On the dorsum of the foot, in the depression proximal to the first metatarsal space.	Perpendicular insertion 16–26 mm
PC6 (Neiguan)	2 *cun* above the transverse crease of the wrist, on the line connecting Quze (PC3) and Daling (PC7), between the tendon of m. palmaris longus and m. flexor carpi radialis.	Perpendicular or oblique insertion 16–33 mm.
KI3 (Taixi)	Posterior to the medial malleolus, in the depression between the tip of the medial malleolus and tendo calcaneus.	Perpendicular or oblique insertion 16–33 mm
CV4 (Guanyuan)	On the anterior midline, 3 *cun* below the umbilicus	Perpendicular or oblique insertion 33–40 mm. This point is often used for moxibustion for tonification.
CV12 (Zhongwan)	On the anterior midline, 4 *cun* above the umbilicus.	Perpendicular or oblique insertion 33–40 mm.
LR14 (Qimen)	Directly below the nipple, in the sixth intercostal space, 4 *cun* lateral to the anteri or midline	Oblique or subcutaneous insertion 16–26 mm
ST44 (Neiting)	Proximal to the web margin between the second and third toes, at the junction of red and white skin	Perpendicular or oblique insertion 16–26 mm
LR2 (Xingjian)	On the dorsum of the foot, proximal to the margin of the web between the first and second toes.	Perpendicular or oblique insertion 16–26 mm

#### Sham EA control

Participants in the control group will receive sham electro-acupuncture treatment at the same acupoints as the treatment group with a blunt tipped placebo needle named Streitberger placebo needle from Germany [17]. This kind of needle can move inside the handle and appear to be shortened after puncturing without penetrating the skin. Participants will find it difficult to distinguish the placebo needle and real acupuncture needle because of the similar sensation and appearance. The electro-acupuncture device will be connected to points, GV20, GV29, bilateral SP6, and ST36 without any electrical current.

### Outcome measures

The sleep quality will be measured by PSQI scores, data from actigraphy, and dose of hypnotics. Depression symptom is accompanied with insomnia in most patients. So we will adopt PHQ-9 to measure the depression condition in lung cancer patients. QLQ-C30 will be used to assess various aspects related to health, disease, and treatment for cancer patients. The treatment effect will be estimated by these subjective questionnaires and objective data from actigraphy, comprehensively evaluating not only the efficacy of EA on insomnia among lung cancer patients, but also the accompanied symptom and quality of life among cancer patients, which seems more meaningful in clinical practice.

#### Primary outcome measure

The primary outcome will be the mean changes in Pittsburgh Sleep Quality Index (PSQI) in week 8 when compared to the baseline. As the most rigorously validated sleep healthy assessment tool, PSQI is a self-rated questionnaire used to assess sleep quality and disturbance over a 1-month time interval. It consists of 24 items to be rated, 19 of which are self-reported and 5 of which are required secondary feedback from a room or bed partner [18]. The seven components are subjective sleep quality, sleep latency, sleep duration, habitual sleep efficacy (SE), sleep disturbance, use of sleeping medication, and daytime dysfunction. The sum of the individual component scores creates one total score (range 0–21). The higher score indicates a worse sleep quality and more severe sleep disorder and vice versa.

#### Secondary outcome measures

##### Actigraphy

Actigraphy is an objective, non-instrusive method for estimating sleep-wake patterns using activity-based monitoring [19]. Computer-based software is interfaced with devices to provide automatic measurements for certain variables recorded in the actigraphy, such as sleep duration, sleep efficiency, and bedtime onset.

##### Quality of Life Questionnaire Core 30 (QLQ-C30)

Quality of Life Questionnaire Core 30 (QLQ-C30) is designed by the Quality of Life Group from the European Organization for Research and Treatment of Cancer (EORTC), specifically for the purpose of assessing various aspects related to health, disease, and treatment for cancer patients. It is composed of 30 items, divided into five functioning domains, three symptom domains, one domain that evaluates overall quality of life, five single domains, and one separate domain to evaluate financial impact [20]. For the functioning domain and overall quality, the higher scores indicate the better quality of life; conversely, for the symptom domain, a higher score indicates a worse quality of life.

##### Patient Health Questionnaire-9 (PHQ-9)

The Patient Health Questionnaire-9 (PHQ-9), as a 9-item self-administered depression screening and diagnostic tool, is based on the Diagnostic and Statistical Manual of Mental Disorders (DSM-IV) depression symptom criteria. PHQ-9 is used to assess depression conditions during the initial 2 weeks. The final summed scores range from 0 (no depressive symptom) to 27 (all symptoms occurring daily) [21].

#### Other measures

##### Dose of hypnotics

Given participants’ psychological condition, oral intake of hypnotics will be allowed to alleviate their insomnia symptoms. Hypnotics will not be restricted, but the name and dosage of drug must be recorded precisely on CRF, especially when the dosage is increased or decreased.

##### Assessment of safety

Any adverse events (AEs) will be observed, those which are deemed to be unfavorable or unintended signs, symptoms, or diseases occurring due to the acupuncture or the hypnotics intake should be dealt with according to the protocol. The type of AEs and severity will be recorded in the CRF, including continuous needling pain, local hematoma, infection, discomfort, palpitation, or dizziness during and after the treatment. If a severe adverse event (SAE) occurs, they should be reported to the researchers and the Ethics Committee in detail within 24 h after the occurrence and be assessed by the Treatment Emergent Symptom Scale (TESS) for further evaluation and management. And then the Ethics Committee will deliver the solution to DSMB, who retain the right to terminate the trial at any point. Researchers will pay sustained attention to participants who experience any AEs until it has been resolved, especially to those who have withdrawn the trial due to the AEs.

##### Assessment of credibility

In this trial, the credibility assessment questionnaire [22] is particularly applied to assess the reliability and credibility in the controlled trials of acupuncture and other physical therapies. The questions presented to the participants for rating on a 6-point scale are as follows: 1. How confident do you feel that this treatment can alleviate your complaints? 2. How confident would you be in recommending this treatment to a friend who suffered from similar complaints? 3. How logical does this treatment seem to you? 4. How successful do you think this treatment would be in alleviating other complaints?

##### Assessment of the subject blinding success rate

All participants will receive the blinding test twice in week 1 and week 8 to assess the success rate of subject blinding. The question is “When you are volunteered for the trial, you were informed that you have the equal chance of receiving traditional acupuncture and acupuncture-like stimulation treatment. Which one do you think you have received?” Participants should choose one of the three answers: acupuncture treatment, acupuncture-like stimulation treatment, or uncertain.

### Statistical methods

#### Sample size estimation

The calculation of sample size is based on the review of acupuncture for insomnia [10]. Referring to this study, the mean difference of PSQI between two groups is assumed to be 2.0 with standard deviation of 4.36 for the both groups. Through PASS system (version 15.0.5) calculating, a sample size of 202 can provide 90% power to reject the null hypothesis with a significance level of 0.05 using a two-side two-sample T-tests assuming equal variance. Considering the expected dropout rate of 20%, the final sample size is 252, 126 for each group.

#### Data collection and management

Data will be collected in the CRF by the assessors after acquiring the signed consent from participants. To guarantee the consistency of data, two research coordinators will double-enter and check data from CRF once a week. To promote participants retention, and prevent their loss, assessors will make phone calls during a 3-month follow up.

#### Statistical analysis

A full analysis set (FAS) is based on ITT principle that is including all the qualified participants who meet the inclusion criteria, who receive the intervention at least once, and who provide outcome assessment at least once. In statistical analysis, any the missing primary outcome will be replaced by the data from last time point according to ITT principle. A Per-Protocol Set (PPS) will be used to analyze those who completed the trial without a major violation of the protocol [23]. A Safety Analysis Set (SAS) is based on the principle of exposure to observe safety indicators for any participants received the intervention at least once.

All analysis will be performed in SPSS24.0. Continuous data will be represented by average, standard deviation, median, minimum value, and maximum value through *t* test; rank-sum test is used for ranked data, while chi-square test is adopted to analyze categorical data [24]. (1) Statistical description: Continuous data from week 0 to week 20 will be presented as MD ± SD. *t* test will be used to compare the difference from baseline in both groups and compare variations after intervention between two groups. (2) Equilibrium analysis: To measure the equilibrium at baseline, *t* test or chi-square test is used to compare demographic data and other basic data at baseline, including name, age, marital status, occupation, education, course of disease, pathologic typing of lung cancer, previous treatment of lung cancer, smoking, drinking, tea, coffee, exercise, pain level, other diseases, ECOG score, PSQI score, data from actigraphy, QLQ-C30 score, PHQ-9 score, and dose of hypnotics. Frequency (composition ratio) will be adopted to describe categorical data of two groups at all time points. (3) Safety analysis: all occurred adverse events will be recorded in both groups. Numbers or the incidence rate in the two groups will be analyzed by chi-square test.

### Quality control

There may exist some potential protocol deviation in this trial. Firstly, it is a multicenter trial, so it demands more than one acupuncturist and assessors, which may cause treatment bias due to different understanding of blinding method and acupuncture methods from different researchers. Secondly, patients may not be in compliance with the intervention time or assessment time or receive any contraindicate treatment as the protocol stated. Thirdly, due to the different conditions of amounts of lung cancer patients, different hospitals may recruit eligible patients with varying speed, which will also lead to protocol deviation. To address the potential bias and guarantee the quality of this trial, despite of improving patients’ compliance, researchers should also strictly follow the protocol and perform adequate randomization, successful blinding, and concealment. As licensed doctors, all acupuncturists and assessors from three hospitals will be required to receive standard training prior to the beginning of the trial. The training program includes recruitment, interventions, and detailed assessment process. Most of all, they will be trained not to discuss the treatment procedure with the patients. Besides, meetings will be held periodically to monitor progress, and researchers will communicate with each other about the arisen problems during the trial and the best solutions. In addition, an independent Data and Safety Monitoring Board (DSMB) will be established to supervise whether the study design meets the standard guideline, and guarantee the preciseness of this trial. The committee consist of five members in different fields: Professor Lixing Lao in acupuncture from Virginia University of Integrative Medicine, Professor Jijun Wang in psychology from Shanghai Mental Health Center, Professor Ruiping Wang in statistics from Yueyang Hospital of Integrated Traditional Chinese and Western Medicine, and Professor Lixin Wang and Professor Peng Zhang in oncology from Shanghai Pulmonary Hospital, who all have to declare no conflict of interest in this study. DSMB is responsible for monitoring data, identifying problems, examining collected data, and controlling bias. Once they find there existing any problems or adverse events during the trial, they have the right to suspend the trial until the problem has been solved or even terminate it at any point.

## Discussion

Nowadays, acupuncture therapy has been widely used in clinical practice as a popular non-pharmacological alternative treatment for insomnia, as more and more research has shown its efficacy and safety. However, most studies focus on acupuncture for general insomnia and there are fewer high-quality studies about acupuncture on treating CRI among lung cancer patients. Thus, it is expected that this study will contribute to adding more strong evidence for the effectiveness of acupuncture for CRI.

The main purpose of this trial is to present a well-designed multicenter randomized single-blinded sham-controlled trial to evaluate the efficacy and safety of electro-acupuncture for insomnia in patients with lung cancer. However, previous researches related to CRI have some limitations, including inexact inclusion criteria, substandard study design, unpractical methods, and indefinite factors decreasing quality of insomnia among lung cancer patients. So we have made some improvements in this trial. Firstly, we design this trial with two groups, EA and sham EA, to identify whether it is real electro-acupuncture that plays a role in treating insomnia in lung cancer patients or it is just the placebo effect. We will limit the inclusion criteria to specific cancer type, ongoing cancer therapy, and tailor points selection for each patient. Secondly, given participants’ psychological condition, oral intake of hypnotics is allowed to alleviate their insomnia symptoms and ensure that each participant gains the optimum benefit from this trial. Thirdly, we will apply semi-standard point selection, composed of core acupoints and additional acupoints, with reference of acupuncture literature and the acupuncturists’ clinical experiences. From additional acupoints, only four of them will be chosen according to specific symptoms of participants, strictly following standardization of syndrome differentiation and treatment administration of TCM. All acupoints will be located with reference to the International Standard Library of Chinese Medicine [25]. Finally, this trial will apply both objective and subjective outcomes representing sleep quality and the related symptoms among lung cancer patients. Most of the acupuncture studies on insomnia have used only patient-reported outcomes; furthermore, a small number of studies using objective outcomes such as actigraphy to show consistent results on the effects of acupuncture [23]. We intend to compare the difference between objective and subjective outcomes and unearth more thought-provoking questions based on the outcomes in this trial.

Although this trial is designed to address the limitations in previous trials, there still remain potential challenges waiting for us to solve. Firstly, to ensure the successful implementation of protocol, acupuncturists will accept training before the beginning of the trial and prepare to reasonably dispose of patients through systematically studying and mastering detailed protocol, particularly in terms of the manipulation of EA and sham acupuncture device and measurement questionnaires. Secondly, to achieve adequate participant enrollment to target sample size in time, the patients will be recruited from three hospitals in Shanghai: Shanghai Municipal Hospital of Traditional Chinese Medicine (TCM), Shanghai Chest Hospital, and Putuo District Central Hospital, which are all comprehensive hospitals with numbers of lung cancer patients on a daily basis. Oncologists in these hospitals have joint this trial, and they will be responsible to introduce this research to eligible patients and recruit them. We also have made some leaflet, billboard inside hospitals, and recruitment link on the Internet to attract patients’ attention. Thirdly, to ensure compliance from patients and ensure minimal loss to follow-up, we will use the following strategies. Clinical trial publicity brochures will be handed out to all hospitals to popularize clinical trial knowledge. Before assigning informed consent, detailed attentions should be explained to participants, including trial protocol and intervention demands, positive effects, and potential adverse events. After fully consideration, they can assign the informed consents. Besides, we will strengthen the public education of medical knowledge to patients, care for the patients, and improve their quality of life in all relevant aspects. For instance, we will make reasonable arrangements for treatment time and consultation time by phone or e-mail to increase their attendance rate. Besides, free acupuncture treatment will be promised by acupuncturists if the patients are not satisfied with the results of their treatment during the trial [26]. And participants will get adequate transportation allowance if they have completed all the treatment and follow-up. What is more, more flexible methods will be adopted to measure patients during the follow-up period, like calling to ask the questions or making an electronic edition of PSQI through message or other communication software. We have successful recruiting experience in our previous studies related to insomnia whose dropout rates were all low. Therefore, we are confident that we will have low dropout rate in this trial.

It still remains uncertain that how EA improve sleep condition in insomnia patients. However, plenty of studies have already demonstrated the potential physiological mechanism through which EA could provide benefit in insomnia. A review [27] make a comprehensive summary of how acupuncture improves sleep quality through regulating monoamine neural transmitters, inhibitory neurotransmitter, cytokines, and immediate-early gene, which plays a vital role in high-quality sleep. A laboratory trial found EA stimulation can induce the increasing concentration of *β*-endorphin, which might be beneficial of sleep by conducting a rat experiment [28]. Acupuncture can also give a dual-directional balancing regulation of autonomic nervous system, sympathetic nervous and parasympathetic nervous included, which might have a sleep-promoting effect [29]. Although this trial is not designed to run any laboratory tests, the results can also provide sufficient evidence to lay a valid foundation on further experimental researches in laboratory, in which the exact mechanism of acupuncture affecting sleep may be found.

To sum up, in this trial, we will standardize point selection, manipulation, assessment, and therapists’ clinical experience following the CONSORT statement and STRICTA recommendations rigorously. We expect this study will confirm the efficacy and safety of acupuncture on insomnia in patients with lung cancer, contributing to filling the gap of the CRI field and providing a promising curative intervention for lung cancer survivors with insomnia in clinic.

### Trial status

The version number of this protocol is 1.0, dated on 1 April 2019. The clinical trial is in preparation at present. The recruitment will begin on 1 May 2020 and be completed in late 2022 as scheduled.

## Data Availability

Not applicable.

## Abbreviations

EA: Electro-acupuncture
CRI: Cancer-related insomnia
PSQI: Pittsburgh Sleep Quality Index
QLQ-C30: Quality of Life Questionnaire Core-30
PHQ-9: Patient Health Questionnaire-9
TESS: Treatment Emergent Symptom Scale
CBT: Cognitive Behavioral Therapy
TCM: Traditional Chinese Medicine
CONSORT: Consolidated Standards of Reporting Trials
STRICTA: Standards for Reporting Intervention in Controlled Trials of Acupuncture
DSM-V: The Diagnostic and Statistical Manual of Mental Disorders, Fifth Edition
APA: American Psychiatric Association
ECOG: Eastern Cooperation Oncology Group
DSMB: Data Safety Monitoring Board
SE: Sleep efficacy
EORTC: European Organization for Research and Treatment of Cancer
DSM-IV: Diagnostic and Statistical Manual of Mental Disorders
CRF: Case report forms
AEs: Adverse events
SAE: Severe adverse event
FAS: Full analysis set
ITT: Intention-to-treat principle
PPS: Per-Protocol Set
SAS: Safety Analysis Set
ANCOVA: Analysis of covariance
